# Impact of Traditional Food Processing Techniques on Mineral Bioaccessibility in Ghanaian Fermented Millet-Based *Koko* and *Zoomkoom*

**DOI:** 10.3390/foods14122126

**Published:** 2025-06-18

**Authors:** Alhassan Wuni, Francis Alemawor, Felix Charles Mills-Robertson, Evans Frimpong Boateng, James Owusu-Kwarteng

**Affiliations:** 1 Department of Food Science and Technology, College of Science, Kwame Nkrumah University of Science and Technology, Private Mail Bag, Kumasi GH233, Ghana; alhassanawuni@yahoo.com (A.W.); falemavor.cos@knust.edu.gh (F.A.); 2 Department of Biochemistry and Biotechnology, College of Science, Kwame Nkrumah University of Science and Technology, Private Mail Bag, Kumasi GH233, Ghana; microbfc2011@gmail.com; 3 Department of Food Science and Technology, School of Agriculture and Technology, University of Energy and Natural Resources, Dormaa Ahenkro Campus, Sunyani P.O. Box 214, Ghana

**Keywords:** *koko*, *zoomkoom*, minerals, bioaccessibility, phytate

## Abstract

In this study, the impacts of traditional processing on phytates contents, phytate: mineral molar ratios, and the bioaccessibility of calcium, iron, and zinc in three traditional *koko* production units (KP1, KP2, and KP3) and two *zoomkoom* production units (ZP1 and ZP2) products were assessed based on the variations in their traditional processing techniques. The total calcium content of ZP1 was ranked the highest (58.02 mg/100 g, *p* < 0.05) compared to other processed samples. A high total value of iron (17.76 mg/100 g, *p* < 0.05) was revealed among *koko* compared to *zoomkoom*. Whereas KP3 and ZP2 showed the highest (*p* < 0.05) amount of zinc (3.34 mg/100 g). ZP1 showed a calcium bioaccessibility of 6.3% (*p* < 0.05). The iron bioaccessibility was within the average range of 5–30%, with KP1 ranking the highest (21.8%), while ZP1 showed the highest value (42.2%) (*p* < 0.05) in bioaccessibility of zinc among the *zoomkoom* products. The processing techniques adopted caused up to a 56.7% to 76.76% reduction (*p* < 0.05) of phytic acid in the pearl millet, leading to a decrease in the molar ratios of [Ca]:[Phy], [Fe]:[Phy], and [Phy]:[Zn]. However, the phytic acid content varied among the *koko* and *zoomkoom,* corresponding with the varied inhibitory mechanism indices reported. In brief, a positive correlation was shown between the traditional processing techniques, phytate, and in vitro bioaccessibility of minerals, indicating the consumption of *koko* and *zoomkoom* as a good source of functional minerals.

## 1. Introduction

In the wake of mitigating food security challenges, undernutrition, and malnutrition aimed at achieving the United Nation’s 2030 sustainable development zero hunger goal, pearl millet (*Pennisetum glaucum*) has gained recognition for its unique nutritional composition [1,2]. Pearl millet is a primary source of protein and energy for millions of people globally and, most especially, has a long history of providing food and nutritional security for the resource-poor populations [3]. Pearl millet (*Pennisetum glaucum*) is a C_4_ carbon fixation tropical orphan cereal grass, originating from Sub-Saharan Africa, with an extensive present cultivation across Asia and ranks as the sixth highest producing agriculture crop [4,5]. Pearl millet has vast agronomic and economic prospects attributed to nutritional functionalities and sustainable utilization in food and human nutrition coupled with versatile end uses [2,6]. Pearl millet is reported to be nutritionally equal or superior to maize, rice, wheat, and sorghum in terms of vital micronutrients and protein [7]. Furthermore, pearl millet’s nutritional composition encompasses energy (17 MJ/kg), fat or lipids (4.8–7.1 g/100 g), protein (9.5–14.41 g/100 g), dietary fiber (8–11.3 g/100 g), vitamins (thiamin, niacin, riboflavin, and tocopherols), antioxidants, and essential nutrients/minerals (calcium (Ca) (16–46 mg/100 g), iron (Fe) (4–11.2 mg/100 g), phosphorus (P), magnesium (Mg), and zinc (Zn) 2.95–7.1 mg/100 g) [4,5,8,9]. Pearl millet has a low glycemic index (an appropriate choice for celiac disease and diabetic patients) and is non-acid forming and gluten free with high amounts of Fe and Zn [10]. However, the nutrient uptake in biological systems from pearl millet grain is limited owing to the presence of antinutritional factors such as phytates, phenolic compounds/polyphenols, fiber, and tannins [10,11].

Minerals are essential micronutrients required for maintaining the proper metabolic functions in the human body, such as by affecting nerve and muscle functions, the body’s water balance regulations, the proper use of vitamins and other nutrients, and bones’ material buildup [12,13]. Per their presence in foods and human requirements, minerals are categorized into macrominerals (magnesium (Mg), potassium (K), calcium (Ca), phosphorous (P), sulfur (S), and sodium (Na)) and microminerals (iron (Fe), manganese (Mn), selenium (Se), zinc (Zn), and copper (Cu)) [14]. According to Rousseau et al. [15], the fraction of nutrients released from the food matrix and available for absorption is known as bioaccessible, usually determined in vitro, whereas the nutrient fraction absorbed and available for use in physiological functions and storage is referred to as bioavailability and can be determined in vivo. However, antinutrient (phytic acid, polyphenols, components of dietary fiber) and physical barriers (cell wall configuration and breakdown of food matrix surrounding) are reported to be the inherent factors of mineral digestion, bioavailability, and bioaccessibility from the food matrix, although plant-based foods are intrinsically rich in minerals. The mineral’s content, bioaccessibility, release, and bioavailability can be affected or lost irreversibly during the various means of processing food (dehulling, cooking, fermentation, milling, extrusion, thermal processing, germination or malting, and soaking) [13]. However, changes in processing strategies can avert mineral losses and increase the content that enhances nutrient bioavailability.

Phytic acid or *myo-inositol hexakisphosphate* (Ins P_6_) is a natural compound in cereals which functions as a chelating agent and contains six reactive phosphate groups [11]. Phytate serves as a phosphate storage molecule in cereals possessing a high affinity to chelates, mostly Fe^2+^, Zn^2+^, K^2+^, Mg^2+^, Cu^2+^, Mn^2+^, and Ca^2+^, that reduce pearl millet’s bioaccessibility and thus prevent their absorption [11,16]. Variations in diets and food supplementation are reported intervention measures to improve the bioavailability and bioaccessibility of Fe and Zn [11]. Pearl millet food products include porridge, bread, couscous, gruel, dough, beer, and non-alcoholic beverages. Several traditional households processing methods in Africa, such as steeping/soaking, malting, grinding, fermentation, roasting, decortication, and flaking, are utilized to improve the edibility, nutritional value, and sensory attributes of foods [17,18]. These strategies are reported to improve the bioavailability and accessibility of nutrients, decrease/eliminate antinutritional factors, and improve the digestibility of plant-based foods. In Ghana, pearl millet is often processed into fermented products such as Hausa *koko*, *zim buli koko*, *zoomkoom* beverages, and *fura,* which have all undergone commercial [19,20,21,22]. *Koko* is a fermented thin porridge, traditionally consumed as a staple food and, as such, forms the major of diets for children and adults in Ghana. Currently, *koko* has gained popularity and is consumed throughout Ghana and other parts of West Africa as a breakfast food, a delicacy, and a weaning food for infants [23]. *Koko* prepared from ground pearl millet with about one-third pepper, cooked with water and served with sugar, bread, and *akara,* has been reported to have high mineral contents and predicted bioavailability [24]. The fermented *zoomkoom* processing steps are similar to Hausa *koko*. Particularly in relation to the fermentation steps. Fermentation is a long-cherished method of processing that produces numerous benefits, such as improvements in the food safety and nutritive values of food. Fermented food products are patronized and consumed globally for their vital functional roles in human nutrition [8]. Fermentation can be performed spontaneously either with a specific starter culture or spontaneously through the endogenous activities inherent in the raw materials or processing environment. Thus, the transformative effect of fermentation on the nutritional value of millet has been documented [4]. Consequently, the traditional fermentation of pearl millet and other processing techniques may result in products with varied nutritional and organoleptic properties. Amidst the essential functionalities of essential nutrients in human nutrition and health, prevalent nutritional issues associated with grain Fe and Zn deficiencies are reported among infants, young children, women of child-bearing ages, and elderly groups in developing nations due to the low bioavailability and bioaccessibility of cereals such as millet [11]. Furthermore, efforts have been made in our agriculture system to mitigate “hidden hunger” or “micronutrient malnutrition” in poor and developing countries to promote human nutrition and health via novel, classical, and biofortification approaches. Yet, pearl millet, which is a nutrient-dense, “orphan”, and potential food security cereal, is underutilized in many parts of the world. Recent studies have highlighted the effect of different processing methods of pearl millet on its nutrients, phytochemical composition, and microbial quality [1], and there is a database of information on the variability existing in pear millet germplasms (phytate amount and antinutrient and goitrogenic compounds and C-glycosylflavones accumulated in the grain) [10]. However, there is scarce information published on the commercially produced *koko* and *zoomkoom* and their mineral nutrients’ in vitro bioaccessibility. Therefore, this study aimed to bridge this gap by unravelling the effect of traditional processing methods on the mineral bioaccessibility of pearl millet *koko* and *zoomkoom*.

## 2. Materials and Methods

### 2.1. Materials

Pearl millet grain used in this study was a traditional landrace variety commonly grown and consumed in the Northern Region of Ghana. Pearl millet grains and spices (cloves, ginger, and pepper) were obtained from Aboabo market in Tamale, and pearl millet (a single variety with greyish color) was cleansed and washed to get rid of contaminants and pollutants from the source before using it to prepare *zoomkoom* and *koko* for this study. Chemicals and reagents, including acetonitrile, formic acid, nitric acid, hydrogen peroxide, and tetrabutylammonium hydroxide, were purchased from the MercK Group (Darmstadt, Germany). Hexane, phytic acid, and dodecasodium salt hydrate were obtained from the Sigma Aldrich Chem. Co., Ltd. (St. Louis, MO, USA). Pepsin was secured from Qualikem Lifesciences Pvt., Ltd. (Gujarat, India). Ammonium nitrate, glucose, potassium chloride, magnesium sulphate, calcium chloride, manganese sulphate, ferrous sulphate heptahydrate, agar, sodium bicarbonate, ferrous sulphate, sodium chloride, and hydrochloric acid were bought from VWR International, (Leuven, Belgium), and all other reagents and chemicals used were of analytical grade.

### 2.2. Sampling Procedure

**Scheme 1:**
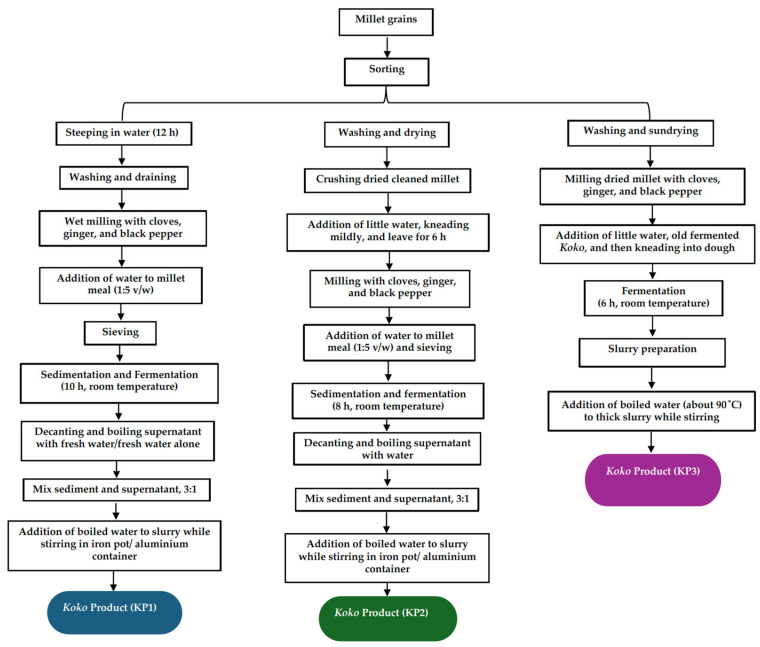
A flow chart showing different processing procedures for the *koko* production. KP1: *koko* processed with steeping (12 h); KP2: *koko* processed by crushing raw millet grains; and KP3: *koko* processed without sieving.

**Scheme 2:**
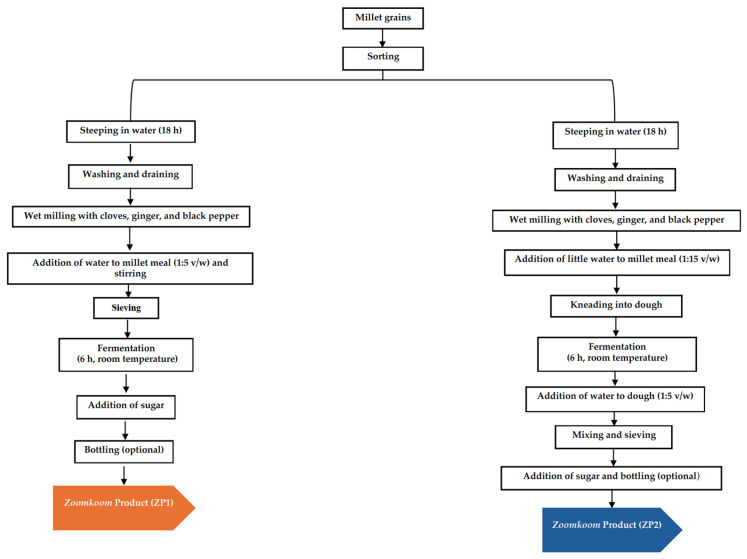
A flow chart showing different processing procedures for the *zoomkoom* production. ZP1: the slurry of the fermented *zoomkoom*; ZP2: the dough of the fermented *zoomkoom*.

The pearl millet grains and spices (cloves, ginger, and pepper) were given to three (3) *koko* and two (2) *zoomkoom* experienced producers, each randomly representing a different traditional processing procedure (Scheme 1 and Scheme 2, respectively) across all treatments. The producers prepared two (2) batches for each of their products, and treatments were compared on consistent endpoints. The samples of *koko* and *zoomkoom* were subjected to phytate and mineral analyses. Sampling was performed thrice. Based on the products’ different processing conditions and duration, the samples were taken aseptically into sterile plastic bottles, allowed to cool to room temperature, and then maintained at 5 °C to degradation prior to analysis.

#### Traditional Production of Pearl Millet into *Koko* and *Zoomkoom*

The formulation in the schematic flows 1 and 2 was adopted in the preparation of *koko* and *zoomkoom*. Five different treatments, each containing primary pearl millet grain and spices, were produced at varying production steps by traditional processors. The treatments were denoted as KP1 (*koko* processed with steeping (12 h), KP2 (*koko* processed by crushing raw millet grains), KP3 (*koko* processed without sieving), ZP1 (pearl millet slurry fermented *zoomkoom*), and ZP2 (pearl millet dough fermented *zoomkoom*) for nutritional quality assessment. The KP1 *koko* production involved the initial steeping of varied quantities of pearl millet grains for an average of 12 h, coupled with onset of fermentation. The steeped grains were then washed thoroughly and repeatedly utilizing running water. Subsequently, the grains were incorporated with spices (chili pepper, ginger, negro pepper, black pepper, and cloves) in a commercial grinding mill and milled into dough. The resulting dough was mixed with unmeasured volume of water to prepare a slurry and sieved using a cheese cloth. The slurry was allowed to ferment spontaneously for 10 h to achieve a sour state. During the fermentation of the slurry, the slurry settles into a precipitate and a supernatant with a foam sitting on top. The foam was scooped off. Furthermore, water was added to the supernatant before boiling per the degree of fermentation. The precipitate portion was divided into two, and the boiled diluted supernatant was poured slowly into one part of the precipitate portion while stirring continuously for consistency. Finally, the other portion of the precipitate was added slowly to obtain *koko* with the desired quality and sensory attributes. The production of KP2 involved a combination of two stages of milling pearl millet grains. First, the pearl millet grain was crushed into pieces following a 6 h moistened treatment aimed at enhancing water absorption to achieve a softened millet grain. Consequently, the softened millet grains were incorporated with spices outlined in KP1 production prior to milling in a commercial grinding mill. The resulting dough was mixed with water to prepare a slurry and sieved using a cheese cloth. The slurry was allowed to ferment spontaneously for about 8 h to turn sour. Therefore, following KP1 preparation description outlined above, desired consistency, quality, and sensory characteristics of KP2 were secured. The production of KP3 considered the following steps. Concisely, pearl millet grains were washed and sundried for 6 h. Spices used in KP1 production were incorporated and subjected to a commercial grinding mill to obtain pearl millet flour. Subsequently, the resulting flour was mixed with water, old fermented *koko*, and then kneaded by hand to prepare dough. The dough was left to ferment spontaneously for 6 h and became sour. Water was added to the dough to form a thick slurry. Afterward, boiled water was used for the KP3 production following the preparation steps outlined in KP1 production. The ZP1 was secured from a fermented slurry of pearl millet grain to achieve *zoomkoom*. Concisely, ZP1 preparation followed the production steps of KP1 above, apart from the cooking stages. For the ZP2 production, the preparation also utilized almost all of the KP1 production steps. Conversely, immediately after grinding, pearl millet grain was mixed with spices and little water was added to the dough and then allowed to ferment for 2–6 h after a period of hand kneading. Thereafter, it was sieved using a standard sieve BS 36, 425 µm mesh aperture. Afterwards, sugar was added for taste. The produced *zoomkoom* was subsequently bottled and refrigerated (±4 °C) before further analysis.

### 2.3. Physicochemical Analysis

The Association of Official Analysis Chemists International (AOAC) method 2011.925.10 (AOAC, 2011) [25] was used for moisture determination. At 105 °C, 5 g of samples were weighed and placed in a hot air oven until a constant weight was achieved. The average initial moisture content was determined based on Equation (1).

(1) $$\%\;moisture\;content=\frac{W2-W3}{W2-W1}\times 100$$

where W1 represents weight of dried Petri dish, W2 represents wet weight of sample and Petri dish, and W3 represents dry weight of sample and Petri dish. The differential method outlined by Karimou et al. [26] with modification was employed to evaluate the dry matter (DM) content. By using Equation (2), the DM content was estimated.

(2) $$\%\;dry\;matter=\frac{M3-M1}{M2-M1}\times 100$$

where M1 represents weight of dried Petri dish, M2 represents wet weight of sample and Petri dish, and M3 represents dry weight of sample and Petri dish.

#### 2.3.1. pH

The desktop pH meter (FE-20, Metter Toledo, Zurich, Switzerland) was employed to determine the pH of samples. Before measurement, the pH meter was calibrated with a buffer (pH 4, 7, and 10).

#### 2.3.2. Total Acidity (Ta)

A modified method of Feng et al. [27] and Paul et al. [28] was adopted to determine total titratable acidity. Concisely, a known mass of samples (*koko* and *zoomkoom*) was diluted 10 times and titrated against 0.5N NaOH using phenolphthalein indicator. The observation of a pale pink color marked the endpoint and was recorded as such. The formula below was used to estimate percent of lactic acid.

(3) $$\%\;lactic\;acid=\frac{\left(titre\times normality\;of\;titrant\times 90.08\right)}{weight\;of\;the\;sample}\times 100$$

### 2.4. Analytical Method

#### 2.4.1. Phytate Extraction and Determination

The pearl millet flour, *zoomkoom,* and *koko* were dried at 37 °C for a constant weight using a moisture analyzer. Dried samples were sieved using a 304 stainless steel wire mesh 20 × 20 screen. The protocol outlined by Pandey et al. [29] was adopted with slight modification for both liquid-phase and solid-phase phytate extraction. Whereas the reverse phase HPLC method per Lehrfeld [30] was employed for phytate analysis with changes to the extraction process, mobile phase, flow rate, and wavelength of quantification.

#### 2.4.2. Determination of Total Minerals

Briefly, 1 g of sample was placed in a microwave digestion system (Milestone MA079, Sorisole, Italy), and then 5 mL of 65% HNO_3_ and 3 mL of 30% H_2_O_2_ were added. The required parameters—170 °C, 1000 watts, and 50 bar for 50 min (25 min for two runs)—were entered for the operation of the microwave digester, and the samples were digested within an hour and allowed to cool. The digest was transferred into graduated centrifuge tubes and diluted to the 25 mL mark with deionized water. The Atomic Absorption Spectrophotometer (Varian 240FS, Mulgrave, Victoria, Australia) equipped was used to determine the total minerals (Ca: 315 nm, Fe: 240 nm, and Zn: 206 nm) content. Each batch of samples had a blank sample and a certified reference material as a quality control.

#### 2.4.3. Bioaccessibility of Minerals

The methods described by Chiocchetti et al. [31] and Pupin et al. [32] with slight modifications were used for in vitro digestion. Pearl millet flour, *zoomkoom,* and *koko* samples were dried at 37 °C until a constant weight was achieved. A total of 5 g of each dried sample was added to 100 mL of basal saline solution consisting of 140 mmol/L NaCl, 6 mmol/L CaCl_2_, and 5 mmol/L KCl in a 250 mL conical flask. Next, 4 mL of α-amylase was added to this solution. The mixture was shaken for 10 min to achieve homogeneity. A 1 M HCl was used to adjust the pH to 2. This was followed by the addition of 3 mL of pepsin (1.6 g in 10 mL of HCl 0.1 M) solution to simulate the peptic phase. The mixtures were then incubated for 2 h at 37 °C in a shaking water bath, after which the assay was kept in ice bath to stop the gastric digestion. Prior to the enteric digestion step, the pH was raised to 7 by drop-wise addition of 1 M NaHCO_3_. Deionized water was added to make it 40 mL. Then, the simulation continued with addition of 10 mL of the pancreatin–bile salt (0.4% of pancreatin (*w*/*v*) and 2.5% of bile extract (*w*/*v*) in NaHCO_3_ 0.1 mol/L (37 °C, 2 h)). To stop the simulation of the enteric digestion, the samples were kept in ice bath for 10 min. Then the digests were transferred to 50 mL centrifuge tubes and centrifuged at 4000 rpm for 30 min. The supernatant was treated with concentrated nitric acid (65% *w*/*w*) and H_2_O_2_ in a microwave digester. The samples were analyzed and quantified using Atomic Absorption Spectrophotometer (Varian 240FS, Mulgrave, Victoria, Australia).

#### 2.4.4. Percentage of Recommended Nutrient Intake (RNI)

The contribution of minerals (Ca, Fe, and Zn) of samples among selected target groups (children, pregnant, and lactating mothers were assessed based on the procedure outlined by WFP/GHS and World Food Programme [33] and Famuyide et al. [34]. A particular nutrient was said to be adequate if it was greater than 70%. Depending on their solid contents, the contributions of 15 g DM (from 100 to 150 g) and 85 g DM (from 500 to 600 g) portion size of *koko* and *zoomkoom* were calculated and expressed in percentage of their RNI.

#### 2.4.5. Estimation of Phytate: Minerals Mole Ratios

The mole ratio computations described by Igwe et al. [35] and Adeyeye et al. [36] were adopted to evaluate phytate: minerals mole ratios per the formulae below.

(4) $$\left[Ca\right]:[Phytate]=\frac{Calcium\;(mg/100g)/40.08}{Phytate\;(mg/100g)/660}$$

(5) $$\left[Phytate\right]:[Zn]=\frac{Phytate\;(mg/100g)/660}{Zinc\;(mg/100g)/65.38}$$

(6) $$\left[Ca][Phytate\right]:[Zn]=\frac{\left[Phytate\;(mg/100g)/660\times [Calcium\;(mg/100g)/40.08\right]}{\left[Zinc\;(mg/100g)/65.38\right]}$$

(7) $$\left[Fe\right]:[Phytate]=\frac{Iron\;(mg/100g)/55.85}{Phytate\;(mg/100g)/660}$$

### 2.5. Statistical Analysis

All the analyses were conducted in duplicate and expressed as means ± standard deviation (SD) of two separate determinations. Microsoft Excel (Version 2019; Microsoft, Redmond, WA, USA), IBM SPSS (Version 20.0 Software for Windows), and OriginLab program (Version 12.5; OriginLab Corporation, Northampton, MA, USA) were employed to complete all analyses. The data generated was subjected to a one-way analysis of variance (ANOVA) and Tukey’s test to determine statistically significant differences (*p* < 0.05), and Pearson’s correlation analysis was used to examine the relationship between parameters.

## 3. Results and Discussion

### 3.1. Physicochemical Properties of Koko and Zoomkoom

**Figure 1:**
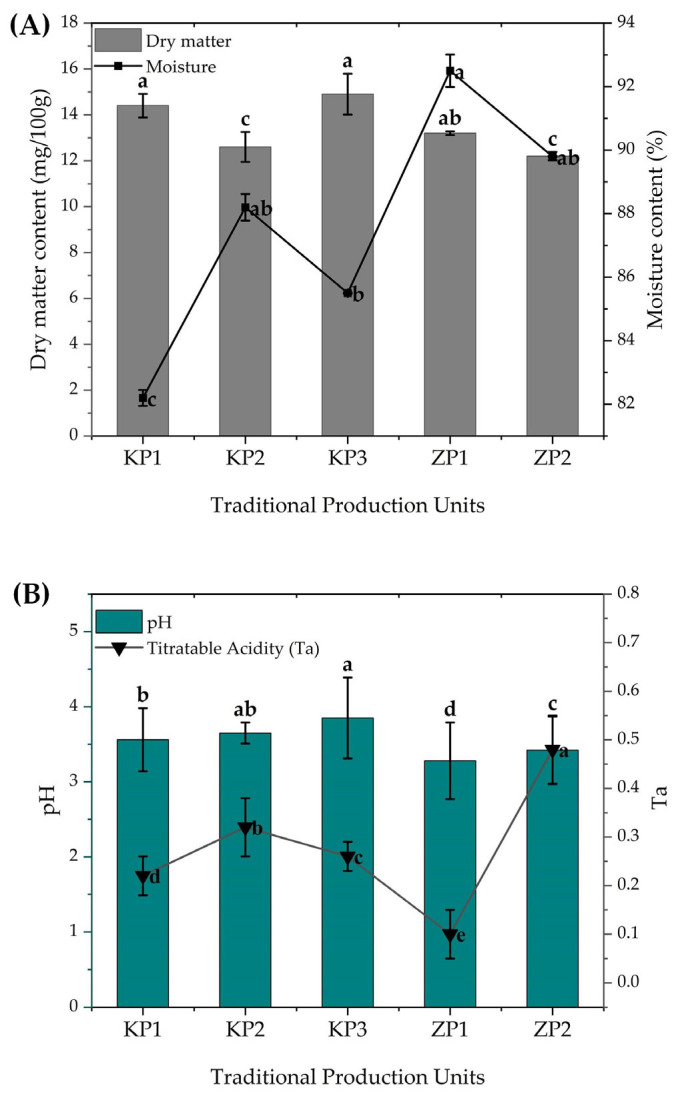
Physicochemical characteristics of *koko* and *zoomkoom*. (**A**) Dry matter and moisture content of *koko* and *zoomkoom*; (**B**) pH and titratable acidity (Ta) of *koko* and *zoomkoom*. KP1: *koko* processed with steeping (12 h); KP2: *koko* processed by crushing raw millet grains; KP3: *koko* processed without sieving; ZP1: slurry of fermented *zoomkoom*; and ZP2: dough of fermented *zoomkoom*. Values are means ± SD (*n* = 3). ^a–e^ Mean values are significantly different (*p* < 0.05).

Figure 1 displays the physicochemical properties of the *koko* and *zoomkoom* derived from various processing procedures. There was a significant difference between the moisture and the DM contents of the KP samples. *Zoomkoom* samples (92.51 ± 0.51 and 89.88 ± 0.02) were found to have a greater moisture content than the *koko* samples. Correspondingly, there was no significant difference between the dry matter content among the processing methods for KP1 and KP3; however, statistical differences existed among processing procedures’ total titratable acidity. The dry matter of *zoomkoom* samples ranged from 12.2 ± 0.18 g to 13.2 ± 0.08 g. Generally, the dry matter contents for *koko* and *zoomkoom* were lower than their water content. The total titratable acidity ranges from 0.10 ± 0.05 of lactic acid to 0.48 ± 0.23 of lactic acid. Generally, the DM contents for all experimental samples were lower than their water content comparatively. Furthermore, the *zoomkoom* samples exhibited higher moisture content values compared to the *koko* samples. In terms of pH, there was a discernible variation in the mean pH values of the *zoomkoom* and *koko* samples. The pH (3.56–3.85) of the fermentation stage was nearly favorable for endogenous and microbial phytases to carry out phytate degradation to release Fe, due to an Fe release–pH mechanism observable in subsequent sections. The 3.56–3.85 pH of the hydrogen ions (H^+^) concentration revealed an acidic nature of the product solubility and the biological availability of minerals such as Fe metals. Thus, the acidic medium dissolved nutrients and kept Fe minerals in the product.

Shi et al. [37] asserted that an increase in Fe levels could be influenced by the pH of the food materials since fermented foods are classified as acidic foods because of their low pH of 4.6. Microbes utilize food products’ starches (carbohydrates and fibers) as an energy source during fermentation [38]. Therefore, the fermentation process influenced the smaller content of the samples’ dry matter. According to Mathlouthi [39], there is a connection between water and foodstuff coupled with the effect of the soluble molecules of the food on the hydrogen bonding in solvent water. Therefore, the revelation of an increased moisture content was relative to the increased water solubility of the *koko* and *zoomkoom* raw materials during processing. The content of acid influences the flavor and palatability of food products [28]. Hence, there was a synergic effect of the pH with the values of the samples’ titratable acidity coupled with a positive preservation functionality.

### 3.2. Phytate Content

**Figure 2:**
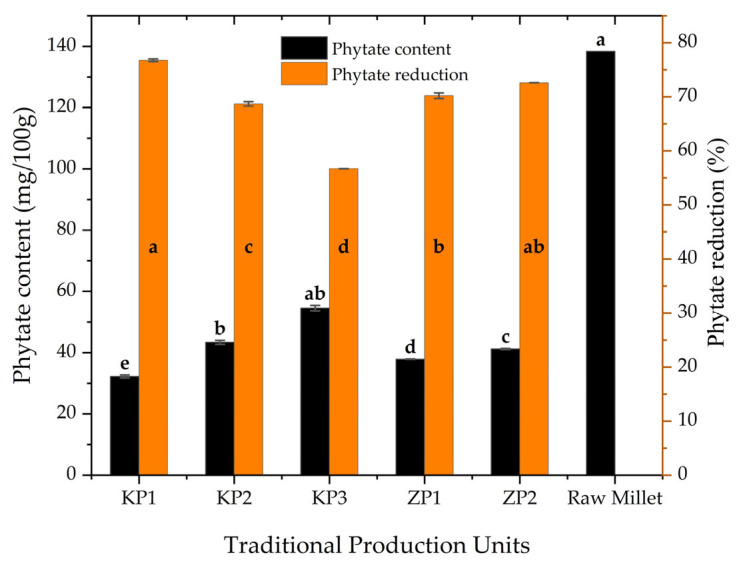
Effect of traditional processing on phytate content and reduction (%) of *koko* and *zoomkoom*. KP1: *koko* processed with steeping (12 h); KP2: *koko* processed with crushing raw millet grains; KP3: *koko* processed without sieving; ZP1: slurry of fermented *zoomkoom*; and ZP2: dough of fermented *zoomkoom*. Values are means ± SD (*n* = 3). ^a–e^ Mean values are significantly different (*p* < 0.05).

Generally, a reduction in the phytate content was observed between the millet and the final *zoomkoom* and *koko* products from all traditional production units irrespective of the processing method employed (Figure 2). This suggests that the employed processing methods of *koko* and *zoomkoom* significantly influenced the decreased amounts of phytic acid values. Compared to other studies, Krishnan & Meera [40] reported the hydrolysis of phytic acid contents among pearl millet varieties. Also, the low phytic acid content could be relative to the pH, species, and soaking duration of samples during processing [41]. Some genetic factors are evidenced by the multidrug resistance-associated protein (tonoplast phytic acid transporter). Thus, protein is stored in the globular crystalloid of legume grains, revealing protein’s relationship with the accumulation of phytic acid via metabolic synthesis [42]. It is worthwhile that the drastic reduction in phytate in samples KP1, ZP1, and ZP2 could be attributed to the application of the soaking (pretreatment) technique, which solubilized the phytic acid salts in water because it was stored in a relatively water-soluble form of phytate (Na, Mg, and K) [43]. Phytate is predominantly present in the aleurone layer and pericarp [44]. Hence, the sieving and fermentation procedures employed in the KP1, ZP1, and ZP2 sample processing removed the bran/pericarp, which serves as storage site for phytate, thereby, resulting in a substantial amount of phytic acid loss. Again, soaking activates endogenous phytases present in grains, resulting in the removal of a significant amount of the phytic acid content in grains [45]. Sihag et al. [46] further described phytate leaching in pearl millet into the soaking water under the influence of a concentration gradient. This phenomenon of the leaching, or the washing out, of phytate into the soaked water, coupled with the pH as a key factor, influences phytic acid’s solubility [47,48]. Fermentation primarily influenced the pH of the fermenting medium, which enhanced microbial activities and endogenous pearl millet phytases to degrade phytate [49]. Conversely, the samples KP2 and KP3 were not soaked during processing. In as much as the KP2 and KP3 sample procedures involved an initial moistening, fermenting, milling, sieving, and slurry fermentation, the procedure did although degrade the phytate, but not as much as observed among the KP1, ZP1, and ZP2 samples in which grain steeping was employed. The pericarp, which serves as a storage site for phytate, formed part of the KP3 final product (*koko*) and hence accounts for KP3’s remarkably high phytate content.

### 3.3. Total Ca, Fe, and Zn

Table 1 presents the mineral contents of the raw pearl millet and treated samples of *koko* and *zoomkoom*. It was evidenced that the treated samples’ employed processing procedures increased the Ca content compared to the raw pearl millet. Also, the total Ca contents in the KP2 and KP3 samples were two times higher than the average total Ca content in the raw pearl millet. Similarly, a significant difference in the Ca content was revealed among all *zoomkoom* samples. The calcium content was six times higher in the steeped and fermented *zoomkoom* (ZP1) and four times higher in fermented dough (ZP2) samples compared to the raw millet. This could be related to the cereal matrix breakdown and the microbial liberation from fermentation interactions of Ca, thus, influencing the Ca to be in an ionized form, thus causing its surge in ZP products. Also, the level of pH impacted the Ca solubilization to an extent. Furthermore, the Fe content of the *koko* was higher compared to the *zoomkoom* and raw pearl millet. Generally, it could be inferred that the adopted traditional processing procedures for treating millet samples influenced the Fe content. There was a marginal increase in the Zn content among the treated samples relative to the sieving and fermentation processing methods employed. A spontaneously fermented slurry prepared from soaked millet increased the Ca content significantly among the *zoomkoom* products. Comparatively, the Ca value of the current study’s raw pearl millet was higher than reported Ca values among Zimbabwean and South African pearl millet [50]. The Fe content was higher compared to those reported values (1.23–2.82 mg/100 g) of the spontaneously fermented finger millet porridge [51], 4.3 mg/100 g pearl millet porridge [52] and 4.13 mg/100 g of unfermented pearl millet porridge [34]. The high Fe level in the pearl millet grains could be related to the high nutrient fertility of the cultivation soils, environmental conditions, and agronomic management [51]. The fortification of millet porridges with micronutrient powder revealed a higher Fe level in the study by Kiio et al. [53] compared to this study’s values. The reduction in the Fe content of *zoomkoom* samples could be due to the longer duration of soaking and shorter duration of fermentation. Aparna et al. [54] reported a reduced total Fe content after 12 h of soaking grains, whereas Afify et al. [55] reported a significant reduction in Fe and Zn in the soaking of sorghum. The Zn content reduced significantly with respect to the KP2 and ZP1 production procedures, but the extent of the reduction in KP3 and ZP2 was non-significant. According to Annor et al. [24], phytate is a major inhibitor of Zn. The findings showed a corroborative Zn content relative to the lower Zn content of the pearl millet reported by Eyzaguirre et al. [56] and Hassan et al. [50]. According to Eyzaguirre et al. [52], Zn is more distributed in the endosperm. Therefore, Zn will leach quite slowly during soaking due to the solubilization synergy with fermentation enzymes [11].

### 3.4. Recommended Nutrient Intake

**Figure 3:**
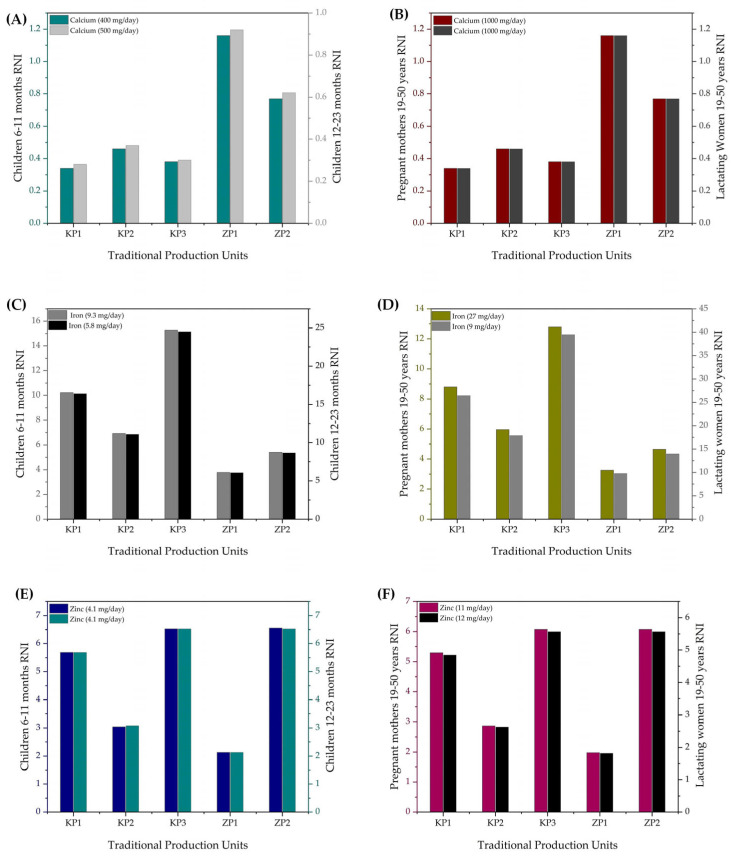
Contribution to Ca, Fe, and Zn requirements of different target groups of *koko* and *zoomkoom*. (**A**) children 6–11 and 12–23 months calcium recommended nutrient intake (RNI) of *koko* and *zoomkoom*; (**B**) pregnant mothers 19–50 years and lactating women 19–50 years calcium recommended nutrient intake (RNI) of *koko* and *zoomkoom*; (**C**) children 6–11 and 12–23 months iron recommended nutrient intake (RNI) of *koko* and *zoomkoom*; (**D**) pregnant mothers 19–50 years and lactating women 19–50 years iron recommended nutrient intake; (**E**) children 6–11 and 12–23 months zinc recommended nutrient intake (RNI) of *koko* and *zoomkoom*; and (**F**) pregnant mothers 19–50 years and lactating women 19–50 years zinc recommended nutrient intake. KP1: *koko* processed with steeping (12 h); KP2: *koko* processed by crushing raw millet grains; KP3: *koko* processed without sieving; ZP1: slurry of fermented *zoomkoom*; and ZP2: dough of fermented *zoomkoom*. Values are means ± SD (*n* = 3).

The contribution of the *koko* and *zoomkoom* to the Ca, Fe, and Zn levels of selected target groups were expected to be 70% of the recommended nutrient intake (RNI) for a serving. A serving of these food products predominantly provided a lower amount of nutrients required by the target groups. However, it was only the lactating mothers’ group that revealed a considerable RNI of >70% for iron (Figure 3). This was because the actual iron content required by this group was quite low (9 mg/day). Even though there was a high bioavailability of Ca, Fe, and Zn in this study, their contribution to nutrient requirements for some targeted groups set by the Ghana Health Service (GHS) could not meet the daily RNI, most especially for children aged 6–23 months and pregnant mothers aged 19–50 years. The difficulty of meeting calcium and iron requirements in *koko,* as well as an enriched *koko*, has been reported [33]. They further reported that calcium, iron, and zinc requirements could not be met in children aged between 6 and 23 months using local foods [33]. In addition to this, de Jager et al. [57] developed food-based dietary guidelines (FBDGs) which cover only 34.3% of the daily RNI and 60.3% of the daily RNI for calcium and iron, respectively, implying the inadequate coverage of FBDGs for the requirements of these nutrients.

### 3.5. Phytate: Mineral Molar Ratios

To determine the samples’ mineral bioavailability, the mole ratios phytate/minerals technique was used per the following recommended critical values: [Fe]:[Phy] > 1.00, [Ca]:[Phy] > 0.24, [Phy]:[Zn] > 15, and [Ca]*[Phy]:[Zn] 0.5 [36]. Statistically, there was a significant difference (*p* < 0.05) in the phytate: minerals molar ratios between the raw millet and treated samples. The molar ratios ranged from 0.04 to 0.19 for [Ca]:[Phy], 0.23–0.79 among [Fe]:[Phy], 1.09–3.73 for [Phy]:[Zn], Ca*[Phy]:[Zn] ranged from 0.47 to 5.39, and then [Fe]:[Zn] 2.18–6.17 for the *koko* and *zoomkoom* samples (Table 2). In general, the decline in phytate/mineral molar ratios was triggered by a drop in phytate during processing and not because of the upsurge in minerals during processing. It is recommended that, for plant-based diets, the [Fe]:[Phy] molar ratio should be <1:1 or better still <0.4:1 for an improved Fe absorption in a cereal-based meal [58]. The influence of processing was relative to the poor Fe bioavailability.

In regard to FAO/IZiNCG, ref. [59] suggested that phytate loses its inhibitory effect on Fe and that Fe becomes soluble and bioavailable when the [Phy]:[Fe] molar ratio is <1.00. What it does suggest is that the Fe absorption from *koko* and *zoomkoom* may not be inhibited significantly by phytate. The results of the [Fe]:[Phy] molar ratio of the present study were generally lower than the [Fe]:[Phy] molar ratios of finger millet porridges from the Hwedza district of Zimbabwe [51]. They alluded that the high [Fe]:[Phy] molar ratios were due to an increased phytate content and a low mineral content. It was however consistent with Eyzaguirre et al. [56], who also reported lower [Fe]:[Phy] molar ratios below the recommended threshold of <1.00 in pearl millet porridges, which were obtained from a combination of processing methods, such as, cooking flour in kanwa, soaking overnight and cooking, and fermentation and cooking. They attributed this to the increase in the level of Fe owing to dry matter losses during soaking and endogenous enzymes converting Fe into a more analyzable form. The molar ratio of [Phy]:[Zn] has been reported as a guide for determining Zn bioavailability [60]. Adeyeye et al. [36] reported <5, 5–15, and >15 to be equivalent to high, moderate, and a problematic Zn bioavailability, respectively. In our study, the [Phy]:[Zn] molar ratio values for all analyzed samples suggested an outstandingly high Zn bioavailability.

Kiio et al. [53] found a very high [Phy]:[Zn] molar ratio of 17.40 in pearl millet porridge, which is not in agreement with the present work. Igwe et al. [61] reported that phytate’s solubility and the amount of the Zn mineral complex in the intestines depend on Ca levels. Thus, Ca has a marked effect on Zn. According to Adeyeye et al. [36], the Ca*[Phy]:[Zn] molar ratio is the best predictor of Zn bioavailability, and a Ca*[Phy]:[Zn] value greater than 0.50 mol/kg would interfere with Zn availability. Therefore, in this study, only one processing procedure had values less than the critical level of 0.50, while the remaining processing procedures for *koko* and *zoomkoom* had greater values, making the Zn in the samples less bioavailable. At this point calcium loses its inhibitory effect on Zn, and the Zn becomes soluble and bioavailable. Concerning the effect of Ca on Zn bioavailability, it is only in Ca*[Phy]:[Zn] that we observed Zn being inhibited by Ca. There is a considerable amount of scientific evidence showing that antinutrients reduce the bioaccessibility of minerals in food [53].

### 3.6. In Vitro Bioaccessibility of Minerals

An in vitro bioaccessibility test was carried out to measure the soluble Fe, Ca, and Zn contents available for absorption in the gut (Table 1). None of the *koko* samples analyzed had soluble Ca; hence, the bioaccessibility of Ca could not be computed. Generally, KP2 recorded a consistently high Fe and Zn bioaccessibility for the *koko*. Regarding Ca, no significant differences existed in the degree of bioaccessibility of calcium between KP1, KP2, and KP3 (*p* > 0.05), despite having significantly different levels of total Ca in the DM basis. This observed contrast could be related to experimental conditions that influenced the dependence on the concentration of total Ca and the amount of soluble Ca under digestion for transport and distribution to cells and tissues in a chemical form for utilization in metabolic functions or for storage.

The average soluble Zn in *zoomkoom* was 0.57 mg/100 g DM and corresponded to a bioaccessibility of 31.4%. The lowest concentration (0.46 mg/100 g DM) was ascribed to ZP1 and the highest value (0.69 mg/100 g DM) to ZP2. Contrarily, ZP1 showed the highest Fe and Zn bioaccessibility (15.2 and 42.2%, respectively) but contained the lowest level of total Fe and Zn in the DM basis. Interestingly, significant differences existed in the amount of bioaccessible Zn and the total Zn between ZP1 and ZP2 (*p* < 0.05). Regarding Ca, significant differences existed in the degree of bioaccessibility of Ca and the total Ca between ZP1 and ZP2 (*p* < 0.05). The number of micronutrients that solubilize in the gut during the digestive process and are made available for absorption is called bioaccessibility. The bioaccessibility of a micronutrient in a regular diet that has undergone standard processing determines staple food crops’ micronutrient status [40]. The bioaccessibility of Fe and Zn in the raw pearl millet used to prepare these products was 11.4% and 6.3%, respectively, corroborated the Fe range of 1.81–16.27% of thirteen studied pearl millet cultivars, while the Zn value was like the bioaccessible values of pearl millet cultivars studied by Krishnan & Meera [40]. The findings from the present work showed that there was no significant change in Fe after following the procedure for the studied *koko* and *zoomkoom* productions. In one instance for the Zn of KP1 and another for the Ca of ZP2, there was a significant difference in the bioaccessible values between the raw millet and the products. As observed from the current studies, the KP2 and KP3 samples revealed bioaccessibility values of Zn which were consistent with the spontaneously fermented pearl millet slurry reported by Gabaza et al. [51], even though the 9.7% value was within the moderate Zn bioaccessibility range but was lower than the results of this work and revealed the moderate Fe bioaccessibility of spontaneous fermented porridge to be 6.1%. Kiio et al. [53] and Famuyide et al. [34] reported bioaccessibility values of 13.2%, 9.98%, and 21.0%, respectively, for Zn. Conversely, the reported lower bioaccessibility values of 0.62 and 4.6 for Fe do not agree with the current study results. Studies by Eyzaguirre et al. [56] also reported an appreciable Fe bioaccessibility value of 14% and 18% in pearl millet porridges prepared from a combination of processing methods (cooking, soaking overnight and cooking, and fermentation and cooking). They indicated that the cooking unit operation had a pronounced effect on the in vitro solubility (IVS) bioaccessibility of Zn possibly due to the complexation of phytate with Fe. They further explained that the reduced IVS of Fe could be due to hydroxyl reactions. Zn bioaccessibility upsurged in cooked “kanwa” up to 48% but dropped to 22% and 18% for overnight soaked and cooked and fermented and cooked samples, respectively. They attributed the increased IVS of Fe and Zn to the rich mineral content of “kanwa”.

### 3.7. Correlation Among Minerals, Phytate, Mineral Bioavailability, and Bioaccessibility

Based on the pooled data from a one-month interval, the correlation analysis was conducted to determine the relationships between the *koko* and *zoomkoom* Ca, Fe, and Zn in Table 3 and Table 4. Phytate is well known to be a strong chelator of divalent cations due to its anionic characteristics, thereby reducing the mineral content in diets. Pearson’s correlation analysis of the results of this present work showed a negative correlation between the phytate and the total Ca and Fe of *koko* and then the phytate and Ca of *zoomkoom*. Furthermore, the phytate content was negatively correlated with the bioaccessibility of the total Ca and Fe of *koko*, then the Zn and Ca of *zoomkoom*, indicating that as phytate levels increased, the total and bioaccessibility of these minerals decreased in the diet. It further showed that the presence and bioaccessibility of these minerals in *koko* and *zoomkoom* depended on phytate, and thus phytate appears to affect their availability and bioaccessibility. Therefore, the decrease in phytate is an important determinant for an improved grain food mineral content and bioaccessibility. However, an unexpected result was encountered as there was a positive, but non-significant correlation between the phytate and total Zn of *koko* and *zoomkoom* and then the phytate and total Fe of *zoomkoom*. The inference was that the anti-nutrient fermentation time and an increase in solubility correspondingly led to an increase in the total Zn of the *koko* and *zoomkoom* and Fe of the *zoomkoom* and vice versa. Also, there was a positive correlation between the phytate and phytate molar ratios of the Ca and Fe of *koko* and the Fe of *zoomkoom*, though it was statistically non-significant, indicating an increase in the phytate content results with the increase in the bioavailability of these minerals. These findings are highly unexpected because phytates typically inhibit Ca, Fe, and Zn contents and bioavailability. What it does suggest is that other factors played major role in the contents and bioavailability of such minerals in the *koko* and *zoomkoom*. Furthermore, the phytate content was non-significantly and positively correlated with the bioaccessibility of the Zn of *koko* and the Fe of *zoomkoom*. Possibly, some antinutritional factors, the reduced pH, and the adopted processing techniques could have counteracted the inhibitory effect of the phytate. The positive correlation between the Ca bioaccessibility, total Ca content, Zn bioaccessibility, and total Zn contents is an indication that higher Ca and Zn contents resulted in a higher Ca and Zn bioaccessibility in *koko* and *zoomkoom*. However, there was a negative correlation between the Fe bioaccessibility and total Fe contents, which is an indication that higher total Fe contents result in a lower Fe bioaccessibility in *koko*. The negative correlation between the bioaccessibility and phytate molar ratio of Ca, Fe, and Zn of pearl millet *koko* and *zoomkoom* indicates that as the bioaccessibility of these minerals (Ca, Fe, and Zn) increases their bioavailability decreases, and vice versa. It thus further suggests that there were complex interactions between the bioaccessibility and phytate molar ratios, where increasing the amount of Ca, Fe, and Zn for absorption does not necessarily translate to the better utilization by the body. The results also showed that the phytate to Fe molar ratio is not a good predictor of the Fe bioavailability of these two millet-based products. A negative correlation has been reported between the Fe bioaccessibility and phytate Fe molar ratio of rice-based products [62]. Thus, they reported that rice-based products’ mineral bioaccessibility was not solely determined by the molar ratio of phytate to iron.

## 4. Conclusions

*Koko* and *zoomkoom* are produced by spontaneously fermenting pearl millet into a porridge or slurry, respectively. *Koko* and *zoomkoom* are traditionally produced in homes or at small-commercial-scale levels and are widely consumed as staple foods, providing a source of energy and nutrients for the population. Consequently, prevalent nutritional issues associated with grain mineral deficiencies are reported among infants, young children, women of a child-bearing age, and elderly groups in developing nations due to cereals’, such as millet, inherent antinutritional factors of mineral digestion, bioavailability, and bioaccessibility. Therefore, this study employed traditional food processing techniques, and the outcome revealed that the bioaccessibility of minerals was generally enhanced for Fe and Zn, but not for Ca, as per the traditional processing methods of processing pearl millet into *koko* and *zoomkoom* and other factors possibly related to cereals’ strong phytate binding. Hence, the various traditional techniques are commendable and have a greater potential for the upscaling and commercialization of *koko* and *zoomkoom*. Also, an enhanced nutritional security for resource-poor populations is revealed, coupled with insights into securing value-added products from nutrient-dense “orphan” pearl millet for convenient and economic gains in the food industries. For this reason, the optimization of the studied processing techniques is recommended to further lower phytate levels and enhance mineral bioaccessibility to address the “hidden hunger” issues.

## Figures and Tables

**Table 1:** The total Ca, Fe, and Zn contents, in vitro bioaccessibility, and molar ratio of the Ca, Fe, and Zn.

Sample	Calcium (Ca)			Iron (Fe)			Zinc (Zn)		
	Total (mg/100 g DM)	IVS (mg/100 g DM)	BA (%)	Total (mg/100 g DM)	IVS (mg/100 g DM)	BA (%)	Total (mg/100 g DM)	IVS (mg/100 g DM)	BA (%)
Raw millet	9.0 ± 0.9 ^aF^	ND	ND	7.9 ± 0.4 ^bD^	0.9 ± 0.5 ^aD^	11.4 ^aD^	2.8 ± 0.7 ^cB^	0.1 ± 0.6 ^bE^	6.3 ^bE^
*koko*									
KP1	17.2 ± 5.1 ^aE^	ND	ND	11.9 ± 1.2 ^bB^	2.6 ± 3.4 ^aA^	21.8 ^aA^	2.9 ± 1.5 ^cB^	ND	ND
KP2	22.9 ± 5.5 ^aC^	ND	ND	8.0 ± 2.2 ^bC^	1.4 ± 1.8 ^aB^	16.9 ^bB^	1.6 ± 0.6 ^cC^	0.4 ± 1.9 ^bD^	23.3 ^aB^
KP3	18.9 ± 8.28 ^aD^	ND	ND	17.8 ± 3.6 ^bA^	1.1 ± 0.7 ^aC^	6.1 ^bF^	3.3 ± 6.9 ^cA^	0.8 ± 0.6 ^bA^	22.4 ^aC^
Average	19.6 ± 4.9 ^aB^	ND	ND	12.6 ± 1.8 ^bA^	1.7 ± 1.6 ^aA^	14.9 ^bA^	2.6 ± 2.4 ^cA^	0.6 ± 1.0 ^bA^	22.9 ^aB^
Range	17.2–22.9	ND	ND	8.24–19	1.1–2.6	6.1–21.8	1.6–2.7	ND-0.8	ND-23.3
CV	27.4	346.4	346.4	69.4/35.2	47.8	47.8	37.7	59.4	59.4
*zoomkoom*									
ZP1	58.0 ± 0.1 ^aA^	3.9 ± 0.0 ^a^	6.8 ^cA^	4.4 ± 1.1 ^bF^	0.7 ± 0.2 ^bF^	15.2 ^bC^	1.1 ± 0.3 ^cD^	0.5 ± 0.4 ^cC^	42.2 ^aA^
ZP2	38.5 ± 8.7 ^aB^	ND	ND	6.28 ± 0.3 ^bE^	0.8 ± 0.6 ^aE^	8.5 ^bE^	3.3 ± 0.9 ^cA^	0.7 ± 0.0 ^bB^	20.6 ^aD^
Average	48.2 ± 4.4 ^aA^	3.9 ± 0.0 ^aA^	8.2 ^cA^	5.3 ± 0.7 ^bB^	0.7 ± 0.4 ^bB^	11.8 ^bB^	2.2 ± 0.6 ^cA^	0.6 ± 0.2 ^cA^	31.4 ^aA^
Range	38.5–58.0	ND-3.9	ND-6.8	3.6–3.8–4.1	0.7–0.8	8.5–15.2	3.2–3.3	0.7–2.1	20.6–42.2
CV	10	0.6	14.6	75	69.7	69.7	77	11.3	ND

KP1: *koko* processed with steeping (12 h); KP2: *koko* processed by crushing raw millet grains; KP3: *koko* processed without sieving; ZP1: slurry of fermented *Zoomkoom*; ZP2: dough of fermented *Zoomkoom*; BA: bioaccessibility; CV: coefficient of variation; ND: non detected; IVS: in vitro solubility; and DM: dry matter. Values are means ± SE (*n* = 3). ^a–c^ Mean values with different superscripts are significantly different (*p* < 0.05) across the minerals, whiles ^A–F^ means values with different superscripts are significantly different (*p* < 0.05) across the treatments.

**Table 2:** Phytate to mineral molar ratios of raw millet, *koko*, and *zoomkoom*.

Processing Procedure	[Ca]:[Phy]	[Fe]:[Phy]	[Phy]:[Zn]	[Ca]*[Phy]:[Zn]	[Fe]:[Zn]
Raw millet	0.95	1.49	4.85	1.09	3.25
KP1	0.12	0.23	1.09	0.47	4.74
KP2	0.12	0.45	2.7	1.54	5.91
KP3	0.19	0.28	1.75	0.82	6.17
ZP1	0.04	0.79	3.73	5.39	4.68
ZP2	0.06	0.51	1.12	1.07	2.18
Critical value	>0.24	>1.00	>15	0.5	4

KP1: *koko* processed with steeping (12 h); KP2: *koko* processed by crushing raw millet grains; KP3: *koko* processed without sieving; ZP1: slurry of fermented *zoomkoom*; and ZP2: dough of fermented *zoomkoom*. Phy: phytate; [Phy]:[Ca]: phytate/calcium mole ratio; [Phy]:[Fe]: phytate-iron mole ratio; and [Phy]:[Zn]: phytate-zinc mole ratio.

**Table 3:** Pearson’s correlations among minerals, between phytate, bioavailability, and bioaccessibility of *koko* prepared from pearl millet.

	T-Ca	T-Fe	T-Zn	B-Ca	B-Fe	B-Zn	Phy	[Phy]:[Ca]	[Phy]:[Fe]	[Phy]:[Zn]
T-Ca	1	0.39 ns	−0.12 ns	0.09 ns	0.39 ns	−0.10 ns	−0.78 **	−0.81 **	−0.75 **	−0.79 **
T-Fe		1	−0.22 ns	0.62 *	−0.05 ns	0.26 ns	−0.36 ns	−0.40 ns	−0.40 ns	−0.23 ns
T-Zn			1	−0.37 ns	−0.48 ns	0.13 ns	0.09 ns	0.16 ns	0.02 ns	0.05 ns
B-Ca				1	−0.13 ns	−0.32	−0.36 ns	−0.34 ns	−0.39 ns	−0.18 ns
B-Fe					1	0.16 ns	−0.01 ns	−0.08 ns	−0.02 ns	−0.20 ns
B-Zn						1	0.13 ns	0.06 ns	0.05 ns	−0.03 ns
Phy							1	0.99 ***	0.95 ***	0.98 ***
[Phy]:[Ca]								1	0.98 ***	0.96 ***
[Phy]:[Fe]									1	0.96 ***
[Phy]:[Zn]										1

ns: non-significant; ns *p* > 0.05; * *p* < 0.05; ** *p* < 0.01; *** *p* < 0.001; Ca: calcium, Fe: iron; Zn: zinc, Phy: phytate; [Phy]:[Ca]: phytate-calcium mole ratio; [Phy]:[Fe]: phytate-iron mole ratio; [Phy]:[Zn]: phytate-zinc mole ratio; T-Ca: total calcium; T-Fe: total iron; T-Zn: total zinc; B-Ca: bioaccessible calcium; B-Fe: bioaccessible iron; and B-Zn: bioaccessible zinc.

**Table 4:** Pearson’s correlations among minerals, between phytate, bioavailability, and bioaccessibility of *zoomkoom* prepared from pearl millet.

	T-Ca	T-Fe	T-Zn	B-Ca	B-Fe	B-Zn	Phy	[Phy]:[Ca]	[Phy]:[Fe]	[Phy]:[Zn]
T-Ca	1	−0.28 ns	−0.52 ns	0.81 *	−0.41 ns	−0.14 ns	−0.91 **	−0.93 ***	0.28 ns	−0.85 **
T-Fe		1	0.79 *	−0.58 ns	0.06 ns	0.49 ns	0.31 ns	0.30 ns	0.82 *	0.32 ns
T-Zn			1	−0.87 **	0.41 ns	0.79 *	0.36 ns	0.34 ns	0.58 ns	0.25 ns
B-Ca				1	−0.32 ns	−0.69 ns	−0.59 ns	−0.61 ns	−0.22 ns	−0.53 ns
B-Fe					1	0.10 ns	0.31 ns	0.31 ns	−0.19 ns	0.07 ns
B-Zn						1	−0.16 ns	−0.16 ns	0.59 ns	−0.22 ns
Phy							1	0.98 ***	−0.26 ns	0.97 ***
[Phy]:[Ca]								1	−0.30 ns	0.97 ***
[Phy]:[Fe]									1	−0.24 ns
[Phy]:[Zn]										1

ns: non-significant; ns *p* > 0.05; * *p* < 0.05; ** *p* < 0.01; *** *p* < 0.001; Ca: calcium, Fe: iron; Zn: zinc, Phy: phytate; [Phy]:[Ca]: phytate-calcium mole ratio; [Phy]:[Fe]: phytate-iron mole ratio; [Phy]:[Zn]: phytate-zinc mole ratio; T-Ca: total calcium; T-Fe: total iron; T-Zn: total zinc; B-Ca: bioaccessible calcium; B-Fe: bioaccessible iron; and B-Zn: bioaccessible Zinc.

## Data Availability

The original contributions presented in the study are included in the article; further inquiries can be directed to the corresponding author.

## Abbreviations

The following abbreviations are used in this manuscript:

RNI	Recommended nutrient intake
BA	Bioaccessibility
CV	Coefficient of Variation
IVS	In vitro solubility
